# Non-indigenous species of Bryozoa from anthropogenic habitats in the Bay of Cádiz (South Iberian Peninsula)

**DOI:** 10.1007/s12526-024-01466-0

**Published:** 2024-09-17

**Authors:** Javier Souto, Oscar Reverter-Gil

**Affiliations:** 1 Institut für Paläontologie, Fakultät für Geowissenschaften, Geographie und Astronomie, Geozentrum, Universität Wien, Josef-Holaubek-Platz 2, 1090 Wien, Austria; 2 Xesús Pousa 10, 6° A, 32001 Ourense, Spain

**Keywords:** *Aeverrillia*, *Hippopodina*, New species, NE Atlantic, Strait of Gibraltar, Spain, NIS

## Abstract

Samples of bryozoans collected from two localities in the Bay of Cádiz with different levels of anthropogenic impact are studied. A total of 25 species was identified, of which 8 are considered non-indigenous (NIS), 12 as native and 5 cryptogenic. A new species of *Hippopodina*, *Hippopodina similis* sp. nov., is here described, and corrections to the diagnosis of the genus are proposed. The species appears to be a recent immigrant in Cádiz, although it could also be present in the eastern Mediterranean. *Aeverrilla setigera* and the genus *Hippopodina* itself are recorded for the first time in the eastern Atlantic. *Anguinella palmata* is recorded for the first time in Spanish waters. *Amathia vidovici* was already recorded in the Iberian Peninsula, but previous records should be confirmed. Other species, such as *Amathia verticillata*, *Biflustra tenuis*, *Watersipora subatra* and *Schizoporella errata*, were already recorded in the Bay of Cádiz very recently.

## Introduction

Introduction and spread of non-indigenous species (NIS), as an accidental or deliberate result of human activities, are one of the greatest threats to marine biodiversity around the world (Mack et al. 2000; Fernández-Romero et al. 2021). The main mechanism for NIS transfer in marine environments seems to be directly related to the current management of worldwide maritime traffic, either associated with the ships’ hulls or by releasing organisms through ballast water (Ruiz et al. 1997; Bulleri and Airoldi 2005; Rocha et al. 2010). Another mechanism for such introductions is aquaculture, either by introducing species of economic interest, but also the unintentional introduction of other species associated with them (Fernández-Pulpeiro et al. 2002; Grosholz et al. 2015; Galanidi et al. 2023). Finally, artificial marine infrastructures such as interoceanic channels have favoured the transfer of certain species (Goren and Galil 2005). For instance, since the Suez Canal was opened in 1869, more than half of the 900 marine alien species recorded in the Mediterranean have probably been introduced from the Red Sea (Zenetos et al. 2012, 2017; Ulman et al. 2017). Most of these NIS first successfully established in the Mediterranean Levantine Sea and then tended to spread to the western Mediterranean (Galil 2006). Marinas are then hubs for NIS arrival, establishment and spread (Canning-Clode et al. 2013; Ros et al. 2020; Sempere-Valverde et al. 2024). As a result, some species have colonised and dominated this artificial habitat across the globe (Guardiola et al. 2012; Kenworthy et al. 2018; Chebaane et al. 2019).

In this context, bryozoan faunas have been identified as an important contributor to the number of species described as NIS in different habitats. This is because of their life mode as mostly sessile, colonial, filter-feeders inhabiting a variety of different natural and artificial substrates such as rocks, floating pontoons, buoys or ropes (Woollacott and Zimmer 1977; Martha et al. 2021). In the Mediterranean Sea, for example, bryozoans are one of the ten phyla contributing the most to the number of marine alien fauna (Zenetos et al. 2012). In particular, in the Iberian Peninsula, the bryozoan fauna is one of the best known in European waters. Our own unpublished compilation of bryozoan species from Iberian waters, based on dozens of articles published over the last century and a half, and the revision of hundreds of samples—both our own and those in museum collections—has yielded approximately 580 Recent species here (Reverter-Gil and Souto 2023 and unpublished data). Nonetheless, our knowledge is still far from complete, as evidenced by the fact that in the last 6 years, 14 new species and four new records have been reported from these coasts (Ramalho et al. 2018, 2020a, 2020b, 2022; Souto et al. 2018; Reverter-Gil et al. 2019; Reverter-Gil and Souto 2021, 2023). This underlines the continued need for purely taxonomic and faunal works, both as key pillars to develop well-designed and useful biodiversity conservation policies (Wägele et al. 2011; Thomson et al. 2018) and also to detect the artificial introduction of alien species. Accordingly, several introduced species of Bryozoa have recently been detected in Iberian waters, some of which seem to be spreading rapidly along the coasts (César-Aldariz et al. 1999; Fernández-Pulpeiro et al. 2002; Hughes et al. 2008; Ryland et al. 2011; Ulman et al. 2017; Souto et al. 2018; Reverter-Gil and Souto 2019, 2023; Ramalho and Caballero-Herrera 2022; Martaeng et al. 2023).

Here, we study samples collected in two localities with different level of anthropogenic impact in the Bay of Cádiz. Previous data from this area are very scarce, with only few papers recording very few bryozoan species (Barroso 1912, 1917, Harmer 1915, Álvarez 1991, Fernández Pulpeiro et al. 1992, Ryland et al. 2011). One more recent paper (Sempere-Valverde et al. 2024) recorded species from several harbours at the Bay of Cádiz.

## Material and methods

**Fig. 1:**
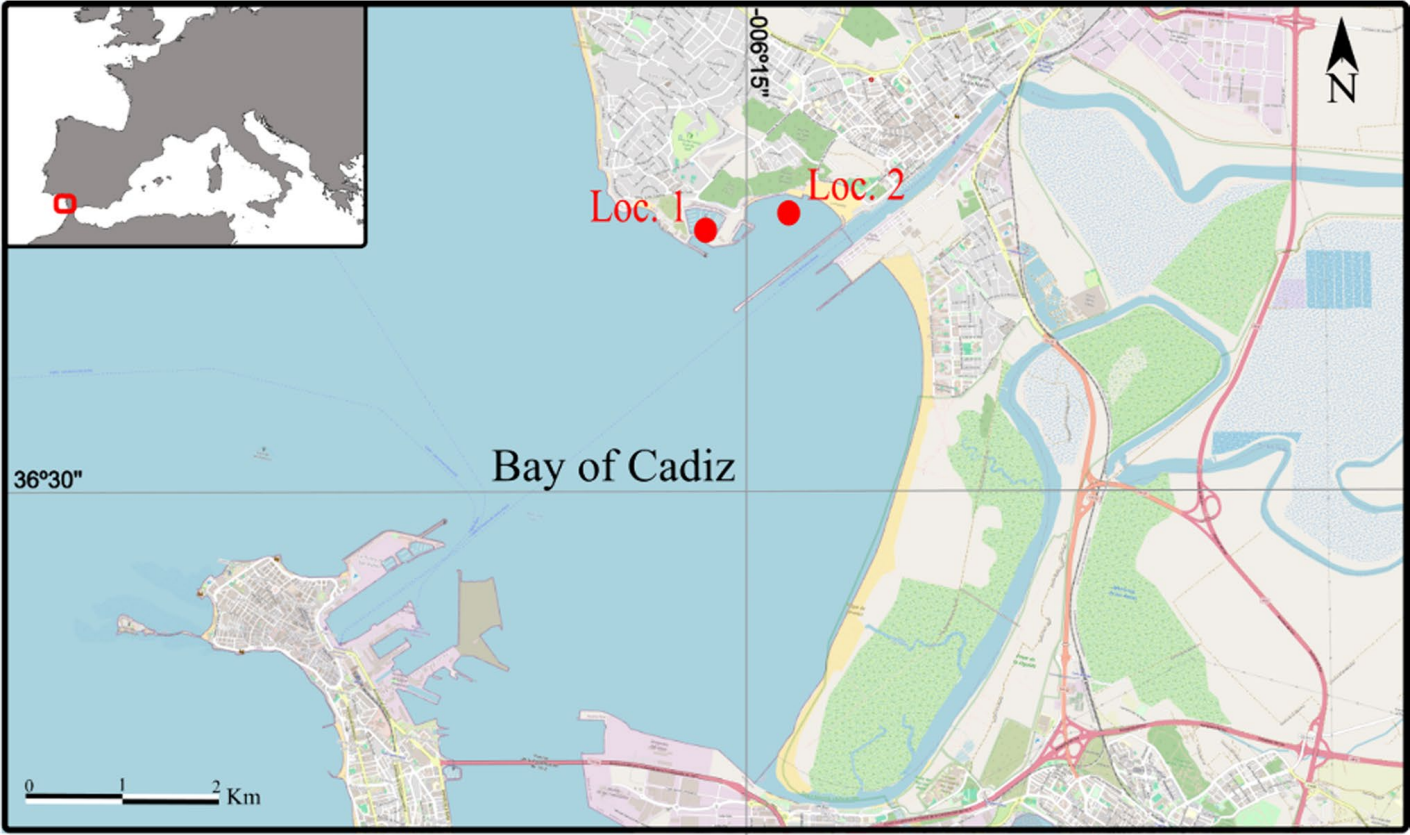
Sampled localities in the Bay of Cádiz. Locality 1, Puerto Sherry; Locality 2, Puntilla Beach (map source: OpenStreetMap and collaborators)

In November 2022, bryozoan assemblages were collected from two localities situated in the “Ensenada del Aculadero”, near the town of El Puerto de Santa María, in the north of the Bay of Cádiz, south Spain (Fig. 1). This small estuary is relatively near the Strait of Gibraltar, which is considered a hotspot of biodiversity (Coll et al. 2010). It is also a major maritime route that is influenced by intense commercial and recreational maritime traffic, which can favour the entry and spread of NIS (Revanales et al. 2022).

Locality 1 is a completely anthropogenic habitat formed by the marina “Puerto Sherry”, where samples were collected by scraping the surface of the plastic and metallic structures forming the floating piers. Locality 2 is situated in “Puntilla Beach”. A rocky outcrop in the centre of this beach represents an intertidal habitat under high anthropogenic pressure from the nearby harbour and other artificial structures and management practices, in addition to the proximity of the city and industries. This outcrop features a high density of the foreign mollusc *Magallana gigas* (Thunberg), which increases the structural complexity of the hard substrate. Shells, algae and small rocks were collected to examine the presence of bryozoans in the lab, while other bryozoan specimens were collected by direct scraping of the rocks.

Samples were fixed in 96% ethanol. Bryozoan specimens were examined in the lab using a Leica MZ12 stereomicroscope. After the first sorting, part of the samples was dried. Selected specimens were cleaned by bleach and dried for study in a FEI Inspect S50 SEM at the University of Vienna. Photographs were taken on uncoated material with a back-scattered electron detector in low-vacuum mode. Optical photos were taken using a Nikon Z6II and a Hirox Rh2000. Measurements were made using the software ImageJ® on optical and SEM photographs.

Selected specimens were sent to the Museo de Historia Natural da Universidade de Santiago de Compostela (MHNUSC).

## Results

### Bryozoan diversity

A total of 25 species was collected during this study (Table 1). Puerto Sherry presents a clearly lower diversity of bryozoans, with only 10 species (six species considered NIS, two natives and two cryptogenic), whereas in Puntilla Beach, up to 23 species were identified (seven NIS, 12 natives and four cryptogenic). Eight species are present in both localities. Only two are exclusive to Puerto Sherry, but 15 to Puntilla Beach. The most relevant taxonomic data are provided here below.

**Table 1:** Species collected in this work

Species	Locality		Status
	Puerto Sherry	Puntilla Beach	
*Aetea anguina* (Linnaeus, 1758)		x	N
*Aetea truncata* (Landsborough, 1852)		x	N
*Aeverrillia setigera* (Hincks, 1887)		x	NIS
*Amathia gracilis* (Leidy, 1855)		x	N
*Amathia lendigera* (Linnaeus, 1758)*		x	N
*Amathia verticillata* (delle Chiaje, 1822)*	x	x	CRY
*Amathia vidovici* (Heller, 1867)		x	CRY
*Anguinella palmata* van Beneden, 1845		x	CRY
*Bantariella verticillata* (Heller, 1867)		x	N
*Beania mirabilis* Johnston, 1840		x	N
*Biflustra tenuis* (Desor, 1848)*		x	NIS
*Bugula neritina* (Linnaeus, 1758)*	x	x	NIS
*Bugulina avicularia* (Linnaeus, 1758)*	x		CRY
*Bugulina flabellata* (Thompson in Gray, 1848)	x	x	N
*Bugulina stolonifera* (Ryland, 1960)*	x	x	NIS
*Buskia nitens* Alder, 1857		x	CRY
*Chartella papyracea* (Ellis & Solander, 1786)*		x	N
*Cradoscrupocellaria ellisi* (Vieira & Spencer Jones, 2012)		x	N
*Cryptosula pallasiana* (Moll, 1803)	x	x	N
*Filicrisia geniculata* (Milne Edwards, 1838)		x	N
*Hippopodina similis* sp. nov.*	x		NIS
*Nolella dilatata* (Hincks, 1860)		x	N
*Schizoporella errata* (Waters, 1878)*	x	x	NIS
*Tricellaria inopinata* d’Hondt & Occhipinti Ambrogi, 1985*	x	x	NIS
*Watersipora subatra* (Ortmann, 1890)*	x	x	NIS

^*^Recorded previously in the Bay of Cádiz

### Taxonomic account

Class Gymnolaemata Allman, 1856

Order Ctenostomatida Busk, 1852a

Superfamily Aeverrillioidea Jebram, 1973

Family Aeverrilliidae Jebram, 1973

Genus ***Aeverrillia*** Marcus, 1941

***Aeverrillia setigera*** (Hincks, 1887)

(Fig. 2)

**Fig. 2:**
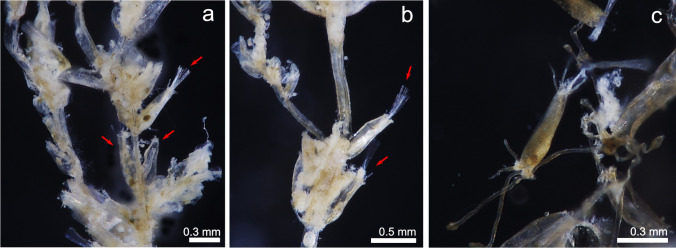
*Aeverrillia setigera;* **a**, **b** View of a colony growing on *Amathia vidovici*; arrows show some of the autozooids (MHNUSC-Bry 738); **c** Zooid and stolons of a colony detached from the substrate (MHNUSC-Bry 740)

*Buskia *setigera Hincks, 1887: 127, pl. 12, figs 9–13.

*Aeverrillia setigera*: Winston and Hayward 2012: 35, fig. 20F; Vieira et al. 2014a: 507, figs 65–68.

**Material examined**: SOUTH IBERIAN PENINSULA • several colonies growing on hydrozoans, other bryozoans and seaweed; Bay of Cádiz, El Puerto de Santa María, Puntilla Beach; 36.58382°N 06.24653°W; depth: intertidal; 10 November 2022; J. Souto leg.; MHNUSC-Bry 737, 738 (Fig. 2A, B), 739, 740 (Fig. 2C), 741, 743.

**Description**: Colony creeping, consisting of an inconspicuous well-chitinized and very narrow stolon, 0.03 mm wide. Zooids often budded in pairs from short kenozooidal peduncles developed on either side of the main stolon. Zooids strongly chitinized, vase shaped, 0.41 to 0.76 mm long by 0.12 to 0.26 mm wide, brown and presenting 4 long acicular cuticular distal spines. Zooids also attached to the substrate with rhizoid-like projections developed from the basal part. Polypide with a distinct gizzard and eight campylonemidan tentacles. Setigerous collar very long, encircling the tentacular crown and protruding as a stiff tuft from partially retracted zooids.

**Remarks**: *Aeverrillia setigera* was originally described from the Mergui Archipelago (Sea of Andaman, Indian Ocean) by Hincks (1887, as *Buskia setigera*). After the original description, this species was reported on several occasions around the world, showing a very improbable distribution by a natural process: In the Atlantic it was recorded from Brazil (Vieira et al. 2014a; Miranda et al. 2018; Silva 2020) and from the USA (see e.g. Winston and Hayward 2012). There is a nominal record from the Beagle Gulf, northern Australia (Gordon 2009). Harmer (1915, as *Buskia setigera*) considered it a very common species in the area between Indian and Pacific Ocean. *Aeverrillia setigera* was also recorded from the Red Sea (Ostrovski et al. 2011). It was also recorded from the Mediterranean in Port-Saïd, close to the Suez Channel (Hasting 1927 as *Buskia setigera*) and is currently considered a non-indigenous species in the Mediterranean (Zenetos et al. 2010, 2012; Rosso and Di Martino 2016), representing a Lessepsian species. Consequently, the origin of the species is unclear, and it was considered to be cryptogenic by several authors (e.g. Marques et al. 2013; Miranda et al. 2018). According to d’Hondt (2002), the locality where *A. setigera* was recorded in the Mediterranean presents a low salinity (below 18 parts per thousand), potentially indicating its resistance to a wide range of environmental conditions.

In the present study, *A. setigera* was recorded only from Puntilla Beach, growing on hydrozoans and other bryozoans such as *Amathia verticillata* (delle Chiaje, 1822) and *Bugula neritina* (Linnaeus, 1758) covering a *Magallana gigas* bed over the rocky outcrop. *Aeverrillia setigera* was already described as a common species growing on filiform substrates on oyster beds (Wells 1961; Larsen 1985).

The origin of the species in the Bay of Cádiz is unknown, but according to previous data, it is considered to be a non-indigenous species here. One potential source is the translocation in the past of *M. gigas* for aquaculture. Nonetheless, the available data on bryozoans in this area are scarce, hindering determining the time of introduction. *Aeverrillia setigera* could have been present for a long time without being detected.

The present record of *A. setigera* represents the first one for the East Atlantic and European waters as a whole.

Superfamily Arachnidioidea Hincks, 1880

Family Nolellidae Harmer, 1915 (1880)

Genus ***Anguinella*** van Beneden, 1845

***Anguinella palmata*** van Beneden, 1845

(Fig. 3)

**Fig. 3:**
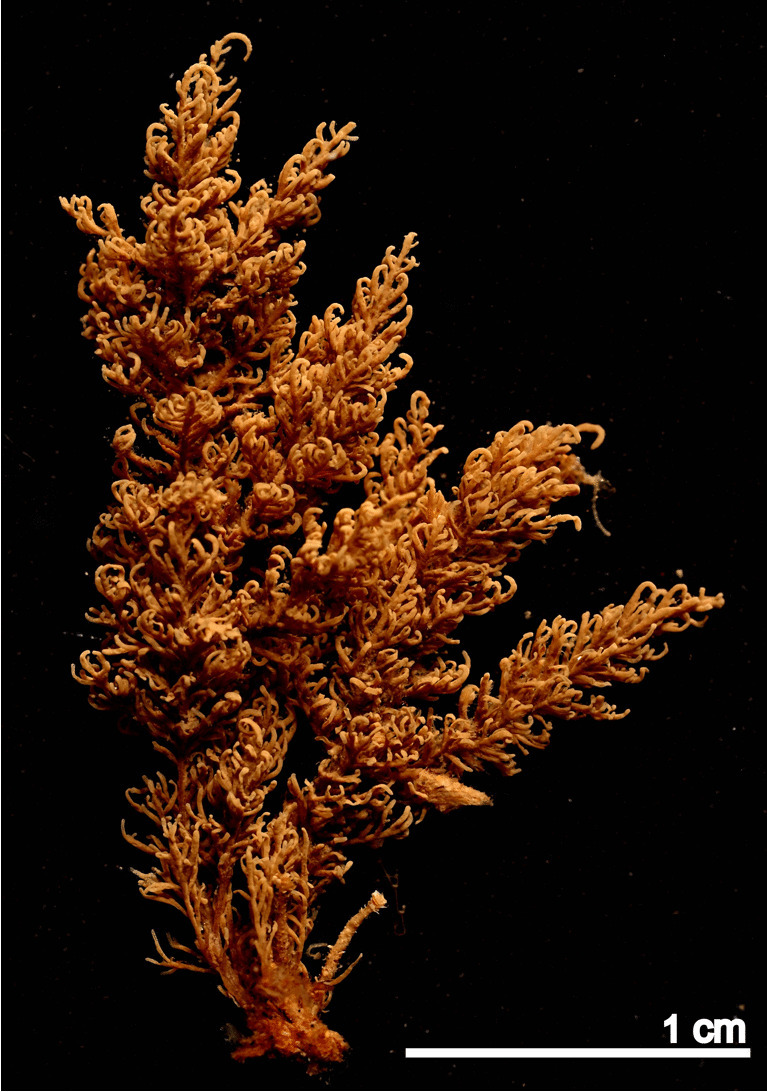
*Anguinella palmata* (MHNUSC-Bry 750). View of a colony

*Anguinella palmata* van Beneden, 1845: 34, pl. 4, figs 18–24; d’Hondt 1983: 43, fig. 25 B; Hayward 1985: 92, fig. 29; Winston and Hayward 2012: 22, fig. 11; Vieira et al. 2014a: 501, fig. 46–48; Reverter-Gil et al. 2016: 129, figs 48, 87D.

**Material examined**: SOUTH IBERIAN PENINSULA • several colonies growing on rock and *Codium* sp.; Bay of Cádiz, El Puerto de Santa María, Puntilla Beach; 36.58382°N 06.24653°W; depth: intertidal; 10 November 2022; J. Souto leg.; MHNUSC-Bry 750 (Fig. 3), 751.

**Remarks**: Accurate descriptions of this species are available in several recent works such as Winston and Hayward (2012), Vieira et al. (2014a) or Reverter-Gil et al. (2016).

*Anguinella palmata* is apparently widely distributed in the temperate North Atlantic: in America from Massachusetts to Brazil (Vieira et al. 2008, 2014a; Winston and Hayward 2012). In Europe, there are scattered records from the south and southwest coast of the British Isles (Hayward 1985), Belgium and the Netherlands (De Blauwe 2009) and Portugal (Souto et al. 2014). However, there are also records from distant points such as California, Peru, Senegal and Congo (d’Hondt 1983, Cook 1985). *Anguinella palmata* is a species of the intertidal zone, mainly inhabiting muddy areas attached to hard substrates, although Marcus (1937) reported it down to 20 m depth in Brazil. However, this material could correspond to a different species, according to Waeschenbach et al. (2015). The species seems to tolerate variations in salinity, so it would be abundant in estuary areas, although it is also present on exposed rocky coastlines (Reverter-Gil et al. 2016).

On the Iberian coasts, *A. palmata* has been previously collected only in an exposed rocky coast at northern Portugal, in the intertidal zone of Baleal (Souto et al. 2014). Consequently, here we present the first record of *A. palmata* in Spanish waters, the second one in the Iberian Peninsula, and the southernmost to date in the European coast. The species was collected in the rocky area of Puntilla Beach, attached to rock and shells of *Magallana gigas*, where it is relatively abundant.

This species is not easily recognisable at first glance as a bryozoan, so its presence may well have gone unnoticed. This makes defining its true distribution difficult. Taking this into account and based on the scarcity of previous data in the area, we cannot presently determine if *A. palmata* is an introduced or a native species in the Bay of Cádiz.

Superfamily Vesicularioidea Johnston, 1838

Family Vesiculariidae Johnston, 1838

Genus ***Amathia*** Lamouroux, 1812

***Amathia vidovici*** (Heller, 1867)

(Fig. 4)

**Fig. 4:**
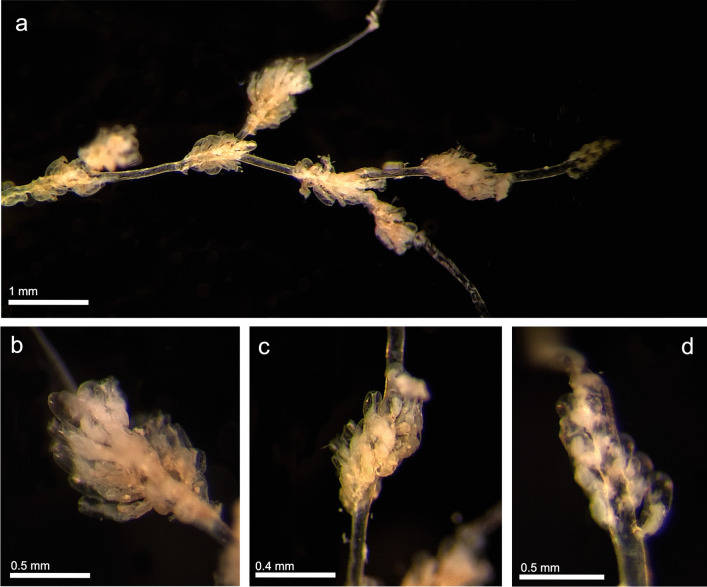
*Amathia vidovici* (MHNUSC-Bry 738); **a** View of a portion of the colony with branching points and several groups of zooids; **b**, **c** Groups of autozooids; **d** Group of autozooids in development at the distal part of the colony

*Valkeria vidovici* Heller, 1867: 128, pl. 5, figs 3, 4.

*Amathia vidovici*: d’Hondt 1983: 65, 67, fig. 35B; Hayward and McKinney 2002: 13, fig. 4B; Reverter-Gil et al. 2016: 178, fig. 67.

**Material examined**: SOUTH IBERIAN PENINSULA • one colony; Bay of Cádiz, El Puerto de Santa María, Puntilla Beach; 36.58382°N 06.24653°W; depth: intertidal; 10 November 2022; J. Souto leg.; MHNUSC-Bry 738 (Fig. 4).

**Remarks**: Accurate descriptions of this species are available in recent works such as Hayward and McKinney (2002) or Reverter-Gil et al. (2016). According to Hayward and McKinney (2002), *A. vidovici* is widely distributed in the Mediterranean, extending in the Atlantic as far as Roscoff on the French coast. It has also been reported from other localities, but its distribution should be revised (Reverter-Gil et al. 2016). The only previous record of this species in Iberian waters was made from Valencia by Barroso (1923), but according to Reverter-Gil et al. (2016), the lack of original material and a complete description in that work makes it impossible to ensure this identification. Another record of *A. vidovici* from Galicia (Fernández Pulpeiro and Reverter Gil 1995; Reverter-Gil and Fernández-Pulpeiro 2001; Souto et al. 2010) possibly actually corresponds to *Amathia citrina* (Hincks, 1877a) (see Reverter-Gil et al. 2016). Therefore, the present record of *A. vidovici* in Cádiz confirms the presence of this species in the Iberian coast.

Family Buskiidae Hincks, 1880

Genus ***Buskia*** Alder, 1857

***Buskia nitens*** Alder, 1857

*Buskia nitens* Alder, 1857: 66, pl. 5, figs. 1, 2; d’Hondt 1983: 57, fig. 1A; Hayward 1985: 152, fig. 53; Reverter-Gil et al. 2016: 195, fig. 77.

**Material examined**: SOUTH IBERIAN PENINSULA • one colony; Bay of Cádiz, El Puerto de Santa María, Puntilla Beach; 36.58382°N 06.24653°W; depth: intertidal; 10 November 2022; J. Souto leg.; MHNUSC-Bry 768.

**Remarks**: Accurate descriptions of *B. nitens* are available in several papers such as d’Hondt (1983), Hayward (1985) or more recently in Reverter-Gil et al. (2016).

*Buskia nitens* is a very difficult species to locate due to the small size of its colonies and the fact that it is often hidden among other organisms. It is thought to be widely distributed, from the Arctic to the tropical seas of the Southern Hemisphere (Reverter-Gil et al. 2016). In the Iberian Peninsula, however, it had been previously collected only in the northwest: Galicia (Reverter-Gil and Fernández-Pulpeiro 2001) and Aveiro (Marchini et al. 2007).

Order Cheilostomatida Busk, 1852a

Suborder Membraniporina Ortmann, 1890

Superfamily Membraniporoidea Busk, 1852b

Family Membraniporidae Busk, 1852b

Genus ***Biflustra*** d’Orbigny, 1852

***Biflustra tenuis*** (Desor, 1848)

(Fig. 5)

**Fig. 5:**
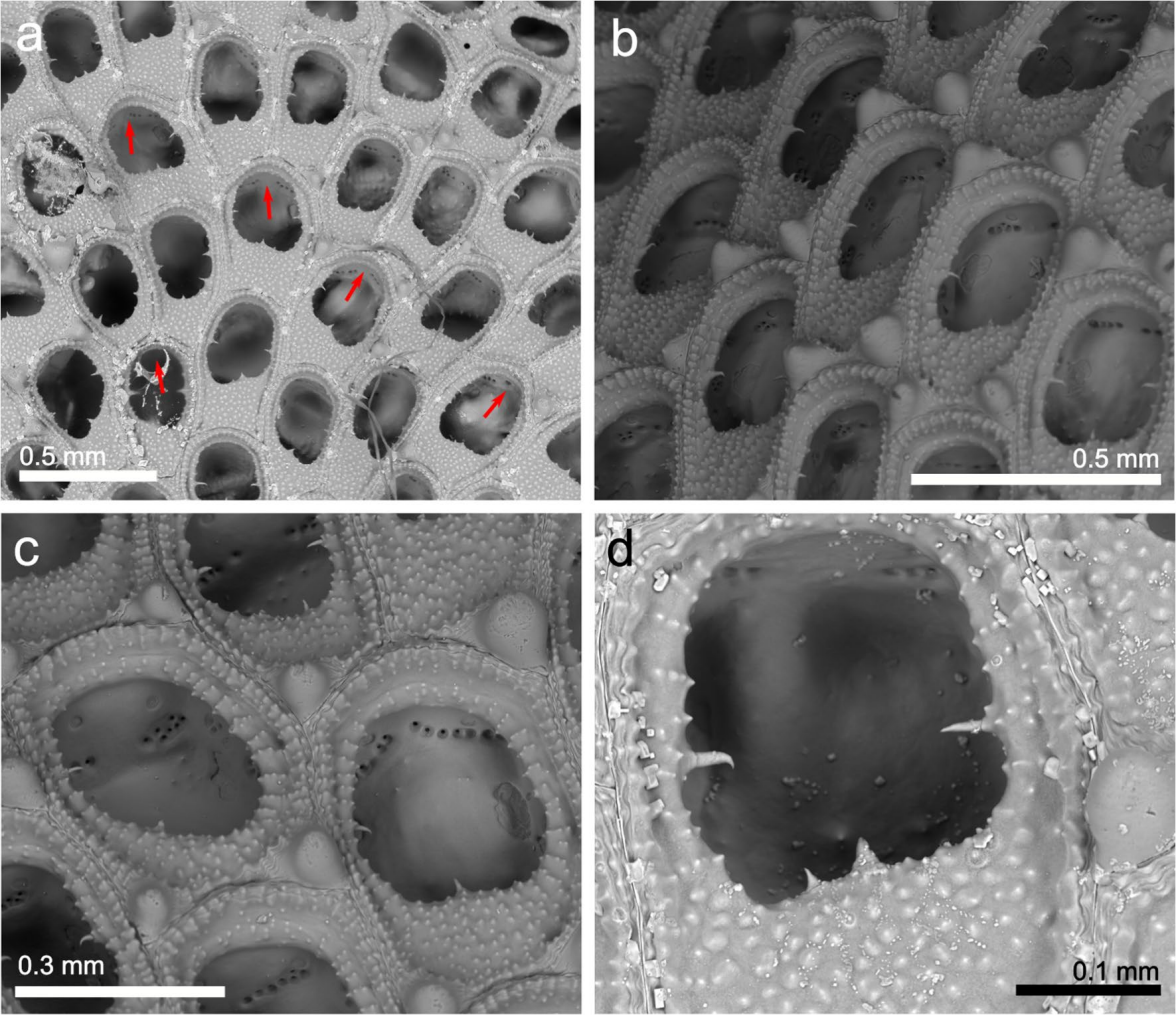
*Biflustra tenuis* (MHNUSC-Bry 755); **a** Frontal view of the colony showing the distal budding forming linear series of autozooids and alternatively the double distal budding (arrows); **b** View of zooids with well-developed proximal gymnocystal tubercles; **c** Distal mural septula on zooids; **d** Close view of the granular cryptocyst and the opesia edge with sharp lateral and proximal denticles

*Membranipora tenuis* Desor, 1848: 66.

*Membranipora tenuis*: Winston and Hayward, 2012: 47, Figs. 26, 29A.

*Biflustra tenuis*: Almeida et al. 2018: 1474, Fig. 8a–d.

**Material examined**: SOUTH IBERIAN PENINSULA • several colonies growing on rocks; Bay of Cádiz, El Puerto de Santa María, Puntilla Beach; 36.58382°N 06.24653°W; depth: intertidal; 10 November 2022; J. Souto leg.; MHNUSC-Bry 747, 755 (Fig. 5), 756 • Several fragments; Bay of Cádiz, Puerto America; March 2017; J. Guerra-García leg; MHNUSC-Bry 754.

**Description**: Colony encrusting, unilaminar, composed of linear series of rectangular to quadrangular autozooids, 0.46 to 0.57 mm long by 0.27 to 0.38 mm wide, with distal end rounded and proximal margin concave, limited by distinct grooves, with prominent, well calcified and coarsely granular lateral walls. Gymnocyst very reduced, so the membranous area occupies practically the entire frontal surface of the zooid, showing in its distal region the operculum provided with a dark-coloured marginal sclerite. Thick and conspicuous gymnocystal tubercles sometimes present at both proximal corners. Cryptocyst surrounding the opesia laterally and proximally, reduced or absent distally; heavily calcified, granular to nodular, with serrated margins, proximal cryptocyst slightly sloping down toward the opesia; two sharp lateral denticles often positioned at the mid-length of the opesia, with an additional single medial denticle in the proximal edge of the opesia; other marginal denticles may also be present. Distal transverse walls with uniporous mural septula, lateral transverse walls with multiporous mural septula.

**Remarks**: According to Zabala and Maluquer (1988, as *Membranipora tenuis*), *B. tenuis* seems to be fundamentally circumtropical, but this statement probably needs to be revised. For instance, Gordon (2016) includes *B. tenuis* from the Sea of China, but highlights the need to revise the specimens of many of the species cited there. Other authors (e.g. Winston and Hayward 2012; Almeida et al. 2018) report the species in the West Atlantic, from Massachusetts to Argentina.

In Iberian waters, *B. tenuis* was found 30 years ago in the intertidal zone of El Portil (Huelva, Gulf of Cádiz) under stones, sharing habitat with *Conopeum reticulum* (Linnaeus 1767), with which it can be confused at naked eye (López de la Cuadra 1991; López de la Cuadra and García-Gómez 1994). More recently, Sempere-Valverde et al. (2024) reported as *Biflusta* cf. *tenuis* material collected in 2016 in two locations in the Bay of Cádiz (marinas of Viento de Levante and Elcano). We have not been able to study the original material, but we have studied other samples collected by the same team in the area in 2017 (see Material examined here above), and we believe that it really belongs to *B. tenuis*.

Suborder Flustrina Smitt, 1868

Superfamily Buguloidea Gray, 1848

Family Candidae d’Orbigny, 1851

Genus ***Cradoscrupocellaria*** Vieira, Spencer Jones & Winston, 2013

***Cradoscrupocellaria ellisi*** (Vieira & Spencer Jones, 2012)

(Fig. 6a, b)

**Fig. 6:**
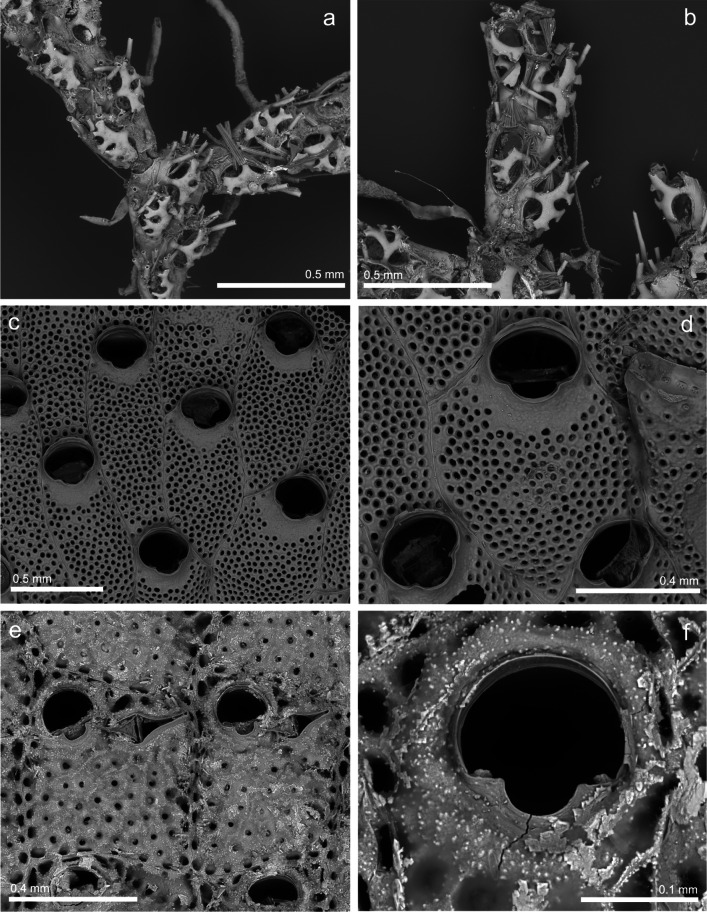
**a**, **b** *Cradoscrupocellaria ellisi* (MHNUSC-Bry 771); **a** View of the branching point; **b** A large frontal avicularia; **c**, **d** *Watersipora subatra* (MHNUSC-Bry 786); **c** View of a colony with several zooids; **d** Detail of an autozooid; **e**, **f** *Schizoporella errata* (MHNUSC-Bry 780); **e** Frontal view of two autozooids with avicularia; **f** Orifice with condyles

*Scrupocellaria ellisi* Vieira & Spencer Jones, 2012: 34, fig. 4, 18–23, 25, 27 (cum syn.).

*Cradoscrupocellaria ellisi*: Vieira et al. 2013: 41, fig. 20; Reverter-Gil et al. 2019: 228, fig. 2d.

**Material examined**: SOUTH IBERIAN PENINSULA • several colonies growing on seaweeds; Bay of Cádiz, El Puerto de Santa María, Puntilla Beach; 36.58382°N 06.24653°W; depth: intertidal; 10 November 2022; J. Souto leg.; MHNUSC-Bry 771 (Fig. 6a, b).

**Remarks**: *Scrupocellaria ellisi* was described by Vieira and Spencer Jones (2012) for several specimens previously identified as *Scrupocellaria reptans* (Linnaeus, 1758). Both species are distinguished by very few characters that are not always readily visible: the presence in *S. ellisi* of smooth rhizoids and stouter scuta with 8–13 stout projections at distal tips versus 6–9 in *S. reptans*, and the size of ooecial pseudopores, which are smaller in *S. ellisi* than in *S. reptans*. In the following year, both species were transferred to the new genus *Cradoscrupocellaria* Vieira et al., 2013.

While the geographical distribution of *C. reptans* is apparently limited to the British Isles, most of its previous records were assigned to *C. ellisi*, a species widespread in the north-east Atlantic. In the Iberian Peninsula, there are many records along the entire coast, made as *S. reptans*, but that must be revised. The presence of *C. ellisi* in Iberian waters was recently confirmed in Galicia (NW of the Iberian Peninsula) (see Reverter-Gil et al. 2019).

Superfamily Smittinoidea Levinsen, 1909

Family Watersiporidae Vigneaux, 1949

Genus ***Watersipora*** Neviani, 1896

***Watersipora subatra*** (Ortmann, 1890)

(Fig. 6c, d)

*Schizoporella aterrima* var. *subatra* Ortmann, 1890: 49.

*Watersipora subatra*: Vieira et al. 2014b: 166, figs. 39–53, 66, 69; Reverter-Gil and Souto 2019: 2738, figs. 2, 3a–c, 7.

**Fig. 7:**
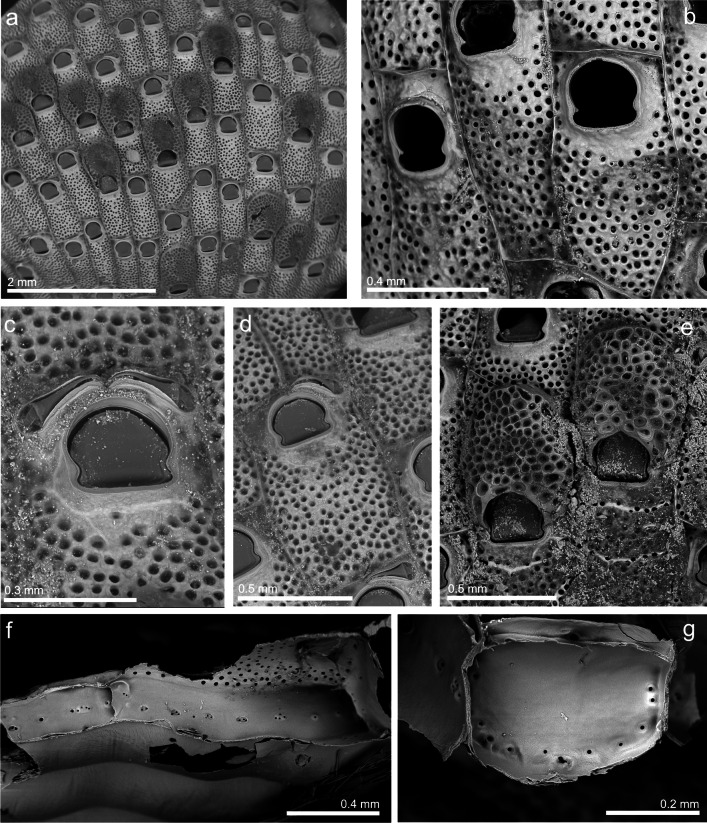
***Hippopodina similis* sp. nov.; a** General view of the colony (MHNUSC 10155–5); **b** Detail of autozooids and orifices (MHNUSC 10155–4); **c** Orifice with two distal avicularia (MHNUSC 10155–5); **d** View of an autozooid with one single distal avicularium (MHNUSC 10155–5); **e** Ovicells (MHNUSC 10155–2); **f** View of lateral wall with multiporous and uniporous septula (MHNUSC 10155–7); **g** Frontal wall with uniporous septula (MHNUSC 10155–7)

**Material examined**: SOUTH IBERIAN PENINSULA • several colonies growing directly on floating pontoons; Bay of Cádiz, El Puerto de Santa María, Puerto Sherry; 36.57947°N 06.25094°W; depth: intertidal; 9 November 2022; J. Souto leg.; MHNUSC-Bry 736 • several colonies growing on rocks and hydrozoans; Bay of Cádiz, El Puerto de Santa María, Puntilla Beach; 36.58382°N 06.24653°W; depth: intertidal; 10 November 2022; J. Souto leg.; MHNUSC-Bry 735, 742, 743, 752, 782, 786 (Fig. 6c, d).

**Remarks**: *Watersipora subatra* was originally described from Japan by Ortmann (1890) but the origin of the species is unclear (see Vieira et al. 2014b for details). It is easily dispersed as a fouling organism on vessels and exhibits a high capacity to grow on artificial substrates subject to anthropogenic disturbance (Reverter-Gil and Souto 2019). The species was recorded for the first time in European waters in 1996 at the coast of Lugo (NW Spain) and has since then spread along the Iberian coast from the Cantabrian Sea (where it was first detected in 2018) to the Algarve (recorded in 2004). As pointed out previously (Reverter-Gil and Souto 2019), however, new samplings are required along the north coast (Cantabrian Sea), the southwest (Gulf of Cádiz) as well as in the Mediterranean, to determine whether the species is still expanding in Iberian waters. Effectively, *W. subatra* has been found very recently for the first time in the Spanish Mediterranean, in the Malaga area and Motril (Ramalho and Caballero-Herrera 2022; Ruiz-Velasco et al. 2022). Interestingly, the species was not detected in the Malaga harbour in 2018, but was detected in 2021, so perhaps, it is a very recent introduction in the area. *Watersipora subatra* was also very recently detected in several localities in the Bay of Cádiz by Sempere-Valverde et al. (2024) based on samples collected in 2016. At that time the species was abundant growing on buoys but only occasionally found on pontoons.

Ramalho and Caballero-Herrera (2022) also reported *Watersipora subtorquata* (d’Orbigny, 1852) in the Malaga area, but the respective photographs evidently belong to *Watersipora souleorum* Vieira, Spencer Jones and Taylor, 2014, another immigrant species already reported from the Gulf of Cádiz, the Strait of Gibraltar and Marseille, but not collected during the present work. *Watersipora souleorum* was already collected in the Malaga harbour in 2018, so its presence in the area could be earlier than *W. subatra*. In fact, *W. souleorum* was collected in Gibraltar (near Malaga) as early as 1894 (see Reverter-Gil and Souto 2019).

Superfamily Schizoporelloidea Jullien, 1882

Family Schizoporellidae Jullien, 1882

Genus ***Schizoporella*** Hincks, 1877b

***Schizoporella errata*** (Waters, 1878)

(Fig. 6e, f)

*Lepralia errata* Waters, 1878: 11, pl. 1, Fig. 9.

*Schizoporella errata*: Tompsett et al. 2009: 2234, Figs. 3A–F, 4A–F.

**Material examined**: SOUTH IBERIAN PENINSULA • several colonies growing on floating pontoons; Bay of Cádiz, El Puerto de Santa María, Puerto Sherry; 36.57947°N 06.25094°W; depth: intertidal; 9 November 2022; J. Souto leg.; MHNUSC-Bry 749, 772, 780 (Fig. 6e, f) • several colonies growing on rock; Bay of Cádiz, El Puerto de Santa María, Puntilla Beach; 36.58382°N 06.24653°W; depth: intertidal; 10 November 2022; J. Souto leg.; MHNUSC-Bry 778, 783.

**Remarks**: According to Tompsett et al. (2009), the ability of *S. errata* to foul man-made structures has clearly mediated its transfer to ports around the world, but the true identities of *S. errata-*like specimens from places distant from the type locality remain open to debate. The high levels of intracolonial morphological plasticity make it likely that the true identity of the Recent *S. errata* complex will require applying molecular techniques.

On the Iberian coast, *S. errata* has been reported previously in various localities, both Atlantic and Mediterranean. These include several locations of the north coast of Galicia (Reverter-Gil and Fernández-Pulpeiro 2001); in Atlantic Andalusia in El Portil (Huelva) and the Island of Tarifa, both relatively near to the Bay of Cádiz (López de la Cuadra and García-Gómez 1988, 1994; López de la Cuadra 1991); in several localities in the Bay of Cádiz in 2016, mainly on buoys but only occasionally on pontoons (Sempere-Valverde et al. 2024); more recently on the Mediterranean coast of Andalusia in the Malaga area (Ramalho and Caballero-Herrera 2022); in the Port of Escombreras (Cartagena) (Morales and Arias 1979); in the Port of Valencia on a ship’s propeller (MHNUSC-Bry 304) (unpublished); and in the Balearic Islands in Mahón (Menorca) (Barroso 1935, 1948, both as *Schizopodrella errata*, Maluquer, 1985, Zabala 1986). In the latter locality we found the species growing on *Mytilus galloprovincialis* Lamarck cultivated in rafts (MHNUSC-Bry 352) (July 2012, unpublished). Accordingly, most of the Iberian records stem from port areas or sites subjected to a strong anthropogenic influence.

Family Hippopodinidae Levinsen, 1909

Genus ***Hippopodina*** Levinsen, 1909

***Hippopodina similis*** **sp. nov.**

http://zoobank.org/7BA7DC36-839A-41D2-BBFB-4BA0DD4D223D

(Fig. 7; Table 2)

**Table 2:** Measurements (in mm) of *Hippopodina similis* sp. nov. (Paratypes)

	Mean	SD	Minimum	Maximun	*N*
Autozooid length	0.862	0.0485	0.772	0.949	22
Autozooid width	0.485	0.0605	0.371	0.633	22
Orifice length	0.232	0.0105	0.218	0.271	22
Orifice width	0.235	0.0101	0.217	0.266	22
Orifice width between condyles	0.189	0.0093	0.173	0.216	22
Avicularium length	0.181	0.0135	0.166	0.192	3
Avicularium width	0.090	0.0035	0.087	0.094	3
Ovicell length	0.511	0.0306	0.448	0.561	12
Ovicell width	0.497	0.0335	0.444	0.560	12

*SD*, standard deviation; *N*, number of measurements

? *Hippopodina feegeensis*: Levinsen 1909: 353 (in part); Corsini-Foka et al. 2015: 358, fig. 4.

*Hippopodina feegeensis*: Sempere-Valverde et al. 2024: 5.

? *Hippopodina* sp. A: Ulman et al. 2017: 16, fig. S2 B, C.

**Material examined**

**Holotype**: SOUTH IBERIAN PENINSULA • several fragments of a colony growing on floating pontoon, in alcohol; Bay of Cádiz, El Puerto de Santa María, Puerto Sherry; 36.57947°N 06.25094°W; depth: intertidal; 9 November 2022; J. Souto leg.; MHNUSC 10154.

**Paratypes**: SOUTH IBERIAN PENINSULA • fragments of a colony growing on floating pontoon, in alcohol; Bay of Cádiz, El Puerto de Santa María, Puerto Sherry; 36.57947°N 06.25094°W; depth: intertidal; 9 November 2022; J. Souto leg.; MHNUSC 10155–1 • several colonies growing on floating pontoons, on SEM stubs; same data as the preceding; MHNUSC 10155–2 (Fig. 7e), 10155–3, 10155–4 (Fig. 7b), 10155–5 (Fig. 7a, c, d), 10155–6, 10155–7 (Fig. 7f, g).

Other material: several fragments; Bay of Cádiz, Puerto America; March 2017; J. Guerra-García leg; MHNUSC-Bry 759.

**Etymology**: From the Latin *similis*, *-e*, meaning similar and referring to the similarities of this species with other *Hippopodina* species (e.g. *H. feegeensis*).

**Diagnosis**: *Hippoporina* presenting large autozooids, 1.8 times as long as wide, with convex frontal wall tuberculate, evenly perforated with numerous circular pores, lacking in the area surrounding the primary orifice. This more or less bell-shaped and as long as wide; anter horseshoe shaped, poster short, of the same width as the anter, with proximal margin straight or very slightly concave. Adventitious avicularium single, very rarely double, but very frequently lacking, positioned disto-laterally to orifice. Ovicellate zooids lack avicularia. Ooecium very large, nearly as long as wide, evenly perforate by large, oval, funnel-shaped pseudopores with rings of concentric calcification. Orifice in ovicellate zooids wider than in autozooids.

**Description**: Colonies of light cream colour in life, multiserial, unilaminar, encrusting floating piers and rocks. Autozooids arranged in linear series, budding only one distal zooid, or occasionally two distal narrow zooids, expanding the colony.

Autozooids large, more or less rectangular, 1.8 times as long as wide, and separated by distinct sutures. Frontal wall slightly convex, tuberculate, evenly perforated with numerous circular pores, lacking in the area surrounding the primary orifice. This slightly raised, more or less bell-shaped and as long as wide; anter horseshoe shaped, poster short, of the same width as the anter, with proximal margin straight or very slightly concave; anter and poster separated on each side by a rounded indentation, within which there is a stout conical condyle.

Communication via about ten uniporous septula positioned all around the margin of the distal wall, grouping in the central area when there are two distal zooids. Lateral walls present a central series of 7 to 10 multiporous or uniporous septula.

Adventitious avicularium single, very rarely double, but very frequently lacking, positioned disto-laterally to orifice; originating laterally and oriented medially, not reaching the axis of the zooid; rostrum short, raised; mandible short, acutely triangular; crossbar complete.

Ooecium very large, nearly as long as wide, evenly perforate by large, oval, funnel-shaped pseudopores with rings of concentric calcification. Ooecium embedded in a concavity on frontal wall of distal zooid. Orifice in ovicellate zooids wider than in autozooids. The operculum closes the orifice. Ovicellate zooids lack avicularia.

Ancestrula not seen.

**Remarks**: Of all the species of the genus, *Hippopodina feegeensis* (Busk, 1884) is certainly the most reported in different parts of the world, including European waters (eastern Mediterranean: Morri et al. 1999; Corsini-Foka et al. 2015). However, as Tilbrook (1999) already demonstrated in its redescription, previous records of the species actually correspond to different species. For example, the records by Levinsen (1909) and Harmer (1957) actually correspond to three different species, two of them new to science (Tilbrook 1999). Unfortunately, the latter author did not include any discussion about these references.

According to Tilbrook (2006), the most useful characters to discriminate between species of the genus *Hippopodina* are the shape and proportions of the primary orifice and the number, shape and orientation of the avicularia, when they exist. *Hippopodina similis* sp. nov. clearly differs from any other species of the genus by several characters:

Although the frontal surface of the zooids is densely perforated, the area surrounding the primary orifice very evidently lacks pores (Fig. 7a–d). This character has not been reported in any other species of the genus. Only Levinsen (1909), in his description of *H. feegeensis*, indicated “…*frontal wall is provided with small, round pores, as a rule densely placed, which may however be wanting on the part round the aperture*.” Moreover, the pores in *H. similis* sp. nov. are proportionally larger compared with those of other species of the genus.

The primary orifice is as long as wide, anter and poster are the same width, and the edge of the poster is straight or very slightly concave (Fig. 7b–d). In *H. feegeensis*, for instance, the poster is 80% the width of the anter.

*Hippopodina similis* sp. nov. has a single (very rarely double), distolateral avicularium, with triangular mandible, but usually absent in most autozooids and in all ovicellate zooids (Fig. 7a–d). In other species, there are two avicularia per zooid, or a single but diversely oriented one, or the whole colony lacks avicularia.

The ooecium is as long as wide, evenly perforate by large, funnel-shaped pseudopores (Fig. 7a, e). This ooecium is similar to that of *Hippopodina viriosa* Tilbrook, 1999, for instance (see Tilbrook 1999: Fig. 2d), but the primary orifice and avicularium are completely different.

Finally, a fossil species has been described in the Iberian Peninsula, *Hippopodina iberica* Pouyet, 1976, from the Pliocene of Las Aguilas (south Spain), so geographically close to Cádiz. However, this species differs from *H. similis* sp. nov. by, among other characters, two distal avicularia also present in the ovicellate zooids and by a very different orifice.

Levinsen (1909) described the presence of uniporous rosette-plates in the genus *Hippopodina* (see also Tilbrook, 1999). Nevertheless, in *H. similis* sp. nov. the septula can be both uniporous and multiporous, with 2 to 4 pores per plate (Fig. 7f, g). No other author seems to have made reference to this character, which perhaps should be included in the diagnosis of the genus *Hippopodina*.

Corsini-Foka et al. (2015) recorded *H. feegeensis* from the Aegean Sea, but the optical figures included in that paper seem to correspond to *H. similis* sp. nov. More recently, Ulman et al. (2017) recorded *Hippopodina* sp. A from Turkey, but again, the figure in that paper seems conspecific with our material. Importantly, in both cases, it would be necessary to study the original material or at least new, more detailed figures to confirm the identifications. The records of *H. feegeensis* in the Mediterranean Sea published by Powell (1969), from Israel, and by Morri et al. (1999), from Greece, are merely nominal records, so their identities cannot be checked.

*Hippopodina feegeensis* was also very recently reported from the Bay of Cádiz by Sempere-Valverde et al. (2024) based on a single sample collected in 2016 on a buoy in Puerto América, but not found in any other locality of the Bay at that time (J.M. Guerra-García, personal communication). We have not been able to study the original material, but we have studied other samples collected by the same team in the area in 2017 (see Material examined here above), and we have verified that they really belong to *H. similis* sp. nov. The rare presence of the species in the Bay of Cádiz in 2016 contrasts with the abundance detected in the present study on floating pontoons, potentially pointing to a recent introduction into the Bay of Cádiz, probably not long before 2016, and a current process of expansion. Although *H. similis* sp. nov. may already be distributed in the Mediterranean, in our opinion, it represents an introduction in the area. This is based on two considerations. Firstly, we know that what Levinsen (1909) reported as *H. feegeensis* actually corresponds to at least three different species, as noted here above. As this author was the only one to report the presence of an imperforate area around the orifice in material of *Hippopodina*, as occurs in *H. similis* sp. nov., it would not be impossible then that Levinsen (1909) had seen material similar to the species described here. Unfortunately, it seems that none of Levinsen’s *Hippopodina* material is preserved. Secondly, most of the Recent species of the genus *Hippopodina* were described from the Pacific, Indonesia or the Caribbean, mostly in tropical or warm waters. The description of new bryozoan species on Iberian coasts, which have been introduced from elsewhere, was also the case, for example, for two other species described from Iberian waters: *Antarctothoa galaica* (César-Aldariz et al., 1999) and *Beania serrata* Souto et al., 2018. These were described as new from Galicia (NW Spain), but probably introduced from other places (see César-Aldariz et al. 1999; Hughes et al. 2008; Souto et al. 2018).

## Discussion

The bryozoan fauna in the area of the Gulf of Cádiz is relatively well known, with a hundred known species in shallow waters close to the coast from Huelva to Tarifa (own unpublished compilation). Nonetheless, our knowledge on the bryozoans in the Bay of Cádiz itself is little better than anecdotal, with only seven species reported until quite recently (Table 3). Moreover, some of these previous records must be considered doubtful regarding their identification (see Álvarez 1991) and/or the exact origin of the record: in some, only “Cádiz” appears as a locality, although that very probably means located in the Bay. To these species, we must add three others that were collected by Rojas Clemente in 1803–1804 on the coast of Cádiz, contained in herbarium sheets currently preserved in the MNCN of Madrid, originally identified as “*Flustra sp. nova*”, and which remained unpublished to date (Table 3). Finally, very recently, Sempere-Valverde et al. (2024) recorded 12 species of bryozoans in harbours in the Bay of Cádiz, growing in buoys and pontoons sampled in 2016. In total, 18 species were known until now in the Bay of Cádiz (Table 3). Of the 25 species identified in the present work (Table 1), 14 are new to the Bay of Cádiz, increasing the number of known species here to 32. On the other hand, in other relatively close areas, outside the Bay, another 18 species have been collected (own unpublished compilation). This brings the total number of species currently known in the surroundings of Cádiz to 46.

**Table 3:** Species previously collected in the Bay of Cádiz

Species	Locality	Reference	Material
*Amathia lendigera*	Cádiz**	Álvarez 1991	MMC 3/M/18
*Amathia verticillata*	Cádiz**	Fernández Pulpeiro et al. 1992	
*Amathia verticillata*	Puerto América, Viento de Levante, San Fernando	Sempere-Valverde et al. 2024	
*Biflustra* cf. *tenuis*	Viento de Levante, Elcano	Sempere-Valverde et al. 2024	
*Bugula neritina*	Cádiz	Ryland et al. 2011	
*Bugula neritina*	Viento de Levante, Elcano, Puerto Sherry, San Fernando	Sempere-Valverde et al. 2024	
*Bugulina avicularia*	Puerto de Santa María	Barroso 1912	
*Bugulina calathus*	Puerto América, Viento de Levante, Elcano, Puerto Sherry	Sempere-Valverde et al. 2024	
*Bugulina calathus minor*	Puerto de Santa María	unpublished (Rojas Clemente)	MNCN 25.03/3575
*Bugulina stolonifera*	Puerto Sherry	Sempere-Valverde et al. 2024	
Bugulina turbinata*	Cádiz**	Barroso 1912, Álvarez 1991	MMC 3/M/52
*Cellaria fistulosa*	Pto. de Santa María and Sanlúcar de Barrameda	unpublished (Rojas Clemente)	MNCN 25.03/3578
*Chartella papyracea*	Pto. de Santa María and Sanlúcar de Barrameda	unpublished (Rojas Clemente)	MNCN 25.03/3578
*Chartella papyracea*	Puerto de Santa Maria and Rota	unpublished (Rojas Clemente)	MNCN 25.03/3576, 3579
*Chartella papyracea*	Puerto América	Sempere-Valverde et al. 2024	
*Hippopodina feegeensis* = *Hippopodina similis* sp. nov	Puerto América	Sempere-Valverde et al. 2024	
*Savignyella lafontii*	Puerto América	Sempere-Valverde et al. 2024	
*Schizobrachiella sanguinea*	Puerto América	Sempere-Valverde et al. 2024	
*Schizoporella errata*	Puerto América, Viento de Levante, Elcano, Puerto Sherry, San Fernando	Sempere-Valverde et al. 2024	
*Schizoporella unicornis*	Cádiz**	Barroso 1917	
*Tricellaria inopinata*	Puerto América, Viento de Levante, Elcano, San Fernando	Sempere-Valverde et al. 2024	
*Triticella flava*	Harbour of Cádiz	Harmer 1915	NHMUK 1911.11.8.31
*Watersipora subatra*	Puerto América, Viento de Levante, Elcano, Puerto Sherry, San Fernando	Sempere-Valverde et al. 2024	

^*^Identification dubious, seeÁlvarez 1991

^**^Exact locality unknown

*MMC*, Museo Marítimo del Cantábrico, Santander

*MNCN*, Museo Nacional de Ciencias Naturales, Madrid

*NHMUK*, National History Museum, London

The increase in the number of species recorded in Puerto Sherry is remarkable, where only five (*B. calathus*, *B. neritina*, *B. stolonifera*, *S. errata* and *W. subatra*) were previously recorded in 2016 (Sempere-Valverde et al. 2024, Guerra-García personal communication), while in the present work we found 10 species, most of them non-indigenous (Table 1). Moreover, Sempere-Valverde et al. (2024) compared the diversity of different organisms between buoys and pontoons in two seasons (summer and winter), concluding that buoys support a higher diversity, which also includes bryozoans. The present work is restricted to pontoon structures (metal and plastic) on one occasion (November 2022), but still yielded a higher diversity, partly contradicting the conclusions of Sempere-Valverde et al. (2024). These differences could reflect the sampling biases or expertise of the respective researchers, but could also be related with the increase of non-indigenous species, favoured by the presence of anthropogenic substrate. Among the 10 species recorded in the marina structures (Table 1), only two (*B. flabellata*, *Cryptosula pallasiana*) are considered native to the region. In the last years the increase in the number of non-indigenous species in the Gulf of Cádiz is considered to be very high. This is probably influenced by the recent anthropogenic alterations of the habitats and by climate change, which facilitates the spread of warm-water biota (González-Ortegón et al. 2020).

The higher number of species at Puntilla Beach (24 spp., Table 1) is consistent with what is expected in a natural habitat. This locality, in spite of have a strong anthropogenic influence (e.g. nearby harbour, intertidal fishing or bathers that visit the area), is natural, with the rocky outcrop in front of the beach. The presence of *M. gigas* changes the substrate surface, and its shells increase the overall complexity by creating small cavities and complex three-dimensional structures. This no doubt favours the microhabitats for bryozoans.

## Conclusions

Of all the species identified in the present paper, *A. setigera* and the genus *Hippopodina* are recorded for the first time in the eastern Atlantic. *Anguinella palmata*, known previously only in northern Portugal along the Iberian Peninsula (Souto et al. 2014), is recorded here for the first time in Spanish waters. *Amathia vidovici* was already recorded in the Iberian Peninsula, but previous records should be confirmed. Nevertheless, its presence in the Bay of Cádiz confirms at least one site along the Iberian coast. *Amathia verticillata* was already recorded in the Gulf of Cádiz in several occasions both in Portugal and the Spanish coast (see e.g. Fernández Pulpeiro et al. 1992, Reverter-Gil et al. 2014). It is currently distributed in the Atlantic and in the Mediterranean coasts of the Iberian Peninsula (Reverter-Gil et al. 2016), although its origin remains unclear.

A new species of *Hippopodina* is here described, and corrections to the diagnosis of the genus are also proposed. These changes pertain to the imperforate area around the orifice and the multiporous septula present in this species, characters not included in the original diagnosis of the genus. Considering that the previous records of *H. feegeensis* and *Hippopodina* sp. A in the eastern Mediterranean are probably synonyms of *H. similis* sp. nov. (see Taxonomic account section), this species could represent a new Lessepsian migrant, potentially arriving at the Bay of Cádiz from the eastern Mediterranean basin around 2016 according with the abundance data of Sempere-Valverde et al. (2024). Remarkably, however, *H. similis* sp. nov. was not recently found in Malaga marinas, on the Mediterranean side of the Gibraltar Strait (Ramalho and Caballero-Herrera 2022), which reinforces the idea of transport associated with boat hulls rather than natural dispersion after its introduction into the Mediterranean.

It may seem surprising that two modest samplings have doubled the number of bryozoan species recorded in the Bay of Cádiz. The description of a new species and the record of several introduced species are also remarkable. This clearly demonstrates that sampling effort and taxonomic studies are still very necessary if we wish to know, and preserve, our biodiversity: we are still very far from reaching that goal.

## Declarations

### Sampling and field studies

All necessary permits for sampling and observational field studies have been obtained by the authors from the competent authorities and are mentioned in the acknowledgements, if applicable.

### Data availability

All data generated or analysed during this study are included in this published article.
